# Selective breeding and selection mapping using a novel wild-derived heterogeneous stock of mice revealed two closely-linked loci for tameness

**DOI:** 10.1038/s41598-017-04869-1

**Published:** 2017-07-04

**Authors:** Yuki Matsumoto, Tatsuhiko Goto, Jo Nishino, Hirofumi Nakaoka, Akira Tanave, Toshiyuki Takano-Shimizu, Richard F. Mott, Tsuyoshi Koide

**Affiliations:** 1 Mouse Genomics Resource Laboratory, National Institute of Genetics, Yata, Mishima, Shizuoka, 411-8540 Japan; 2 Department of Genetics, SOKENDAI (The Graduate University for Advanced Studies), Yata, Mishima, Shizuoka, 411-8540 Japan; 3 Graduate School of Medicine, Nagoya University, Tsurumai-cho, Showa-ku, Nagoya, 466-8550 Japan; 4 Division of Human Genetics, National Institute of Genetics, Yata, Mishima, Shizuoka, 411-8540 Japan; 5 Transdisciplinary Research Integration Center, Toranomon, Minatoku, Tokyo, 105-0001 Japan; 6 Drosophila Genetic Resource Center, Kyoto Institute of Technology, Kyoto, 616-8354 Japan; 7 Genetics Institute, University College London, Gower Street, London, WC1E 6BT UK

## Abstract

Tameness is a major behavioral factor for domestication, and can be divided into two potential components: motivation to approach humans (active tameness) and reluctance to avoid humans (passive tameness). We identified genetic loci for active tameness through selective breeding, selection mapping, and association analysis. In previous work using laboratory and wild mouse strains, we found that laboratory strains were predominantly selected for passive tameness but not active tameness during their domestication. To identify genetic regions associated with active tameness, we applied selective breeding over 9 generations for contacting, a behavioural parameter strongly associated with active tameness. The prerequisite for successful selective breeding is high genetic variation in the target population, so we established and used a novel resource, wild-derived heterogeneous stock (WHS) mice from eight wild strains. The mice had genetic variations not present in other outbred mouse populations. Selective breeding of the WHS mice increased the contacting level through the generations. Selection mapping was applied to the selected population using a simulation based on a non-selection model and inferred haplotype data derived from single-nucleotide polymorphisms. We found a genomic signature for selection on chromosome 11 containing two closely linked loci.

## Introduction

Tameness is one of the major factors selected during domestication^1–3^. Allele frequency changes have been shown to occur over generations in a population of captive wild animals adapting to humans^4^. Genetic loci for tameness in selectively bred rat and fox populations were previously identified by performing genetic analyses using genome-wide association studies (GWAS) and quantitative trait locus (QTL) mapping^5, 6^. Even though attempts have been made to identify genes related to tameness in these animals, limited information about the genetic basis underlying tameness has been clarified. Recent genomic analyses using numerous genetic markers for domesticated and wild animals revealed genetic loci potentially associated with tameness^7, 8^. These studies were focused on measuring selected loci using heterozygosity or population genetics metrics such as *F*_*st*_. However, these approaches were affected by the structures of the populations being compared, which had been affected by uncontrolled migration. Furthermore, little evidence was available for the candidate loci being associated with behaviour because the studies were focused on DNA sequence variation in domesticated animals and few measurements of behavior were made. Because of these shortcomings, new systematic attempts must be made to clarify the genetic basis of tameness in animals.

Tameness has two potential components, a motivation to approach humans and a reluctance to avoid humans^3^. To quantify these components in mice, we previously developed three handling tests, called active tame, passive tame, and stay-on-hand tests^9^. The active tame test is used to evaluate motivation to approach a human hand (active tameness). The passive tame and stay-on-hand tests are used to evaluate reluctance to avoid a human hand (passive tameness). We used these behavioral tests to assess seven laboratory strains of mice established up to a century ago from domesticated mice of European origin (*Mus musculus domesticus*) and ten wild strains, more-recently derived from three major genetically diverse subspecies of mice, (*M. m. domesticus, M. m. musculus*, and *M. m. castaneus*). We found that tameness varied significantly depending on the genetic background of the mice, and the heritability of the 9 traits including both active and passive tameness varied between 0.38 to 0.73 at six weeks old^9^. We found that laboratory strains exhibited significantly more passive tameness than wild strains but that the degree of active tameness in the two groups was not significantly different. These results suggest that selection during domestication of laboratory mice predominantly involved passive but not active tameness. Further studies of active tameness in mice are required to improve our understanding of the genetic basis of tameness.

It is crucial that a mouse stock that exhibits high levels of active tameness is established for use in studies of active tameness because such a strain has not previously been established, and is essential to allow the effects of genes to be examined in future studies. From this point of view, the commonly used QTL mapping method using a F2 hybrid population produced using two different inbred strains will not be suitable for analyzing genetic loci related to tameness. Active tameness is probably regulated by multiple genes, so it is important that genetic heterogeneity and diversity are extended by crossing multiple strains to allow genes related to active tameness to be identified.

In the study described here, we aim to identify genetic loci associated with active tameness through selective breeding of a novel outbred stock, wild-derived heterogeneous stock (WHS), derived from inbred progenitors, followed by genomic analysis of the mouse populations. The study had three stages. We first chose eight wild mouse strains to establish a WHS and studied the genetic characteristics of the founders using genome-wide SNP data. We then established a WHS by crossing the eight strains and selectively breeding for “contacting,” which is strongly associated with active tameness^9^. Finally, we performed selection mapping to identify loci associated with this trait.

## Results

### Phylogenetic analysis of wild and domesticated strains

**Figure 1:**
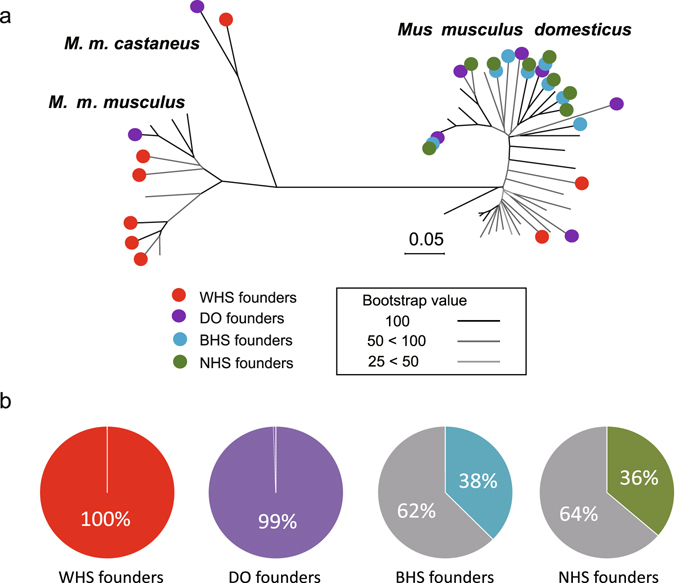
Genetic characteristics of the mouse strains used to establish the wild-derived heterogeneous stock (WHS). (**a**) Neighbor-joining tree constructed using single nucleotide polymorphism (SNP) data from eight WHS founder strains and other inbred strains. The bootstrap test was performed 1,000 times. A total of 10,594 SNPs obtained using the MegaMUGA array were used. Because the SNPs of the array were selected to show genetic differences among *M. m. domesticus* subspecies, the genetic differences among *M. m. musculus* subspecies appear to be smaller than the actual genetic distances. (**b**) Circular charts indicating the percentages of the 10,594 in each stock showing polymorphisms for the eight founder strains. The grey section indicate percentages of SNPs showing no polymorphism.

Eight wild mouse strains, BFM/2Ms (BFM/2), PGN2/Ms (PGN2), HMI/Ms (HMI), BLG2/Ms (BLG2), CHD/Ms (CHD), KJR/Ms (KJR), MSM/Ms (MSM), and NJL/Ms (NJL) were selected to establish the WHS. All of these originated in different countries as founder strains (Table S1), and represent all three major subspecies of *M. musculus*. The genetic characteristics of the WHS were determined by performing genomic SNP analyses using the MegaMUGA array, which covered 77 K SNPs^10^. The genotypes of the eight founder strains were compared with the genotypes of 46 other strains for which data are available from the UNC Systems Genetics at the University of North Carolina (http://csbio.unc.edu/CCstatus/index.py?run=GeneseekMM). After quality control of the SNP data, we chose 10,594 genome-wide SNPs for which genotypes were available for all the strains used in the subsequent analysis. A neighbor-joining (NJ) tree using these SNPs revealed that the WHS founders have different genetic characteristics from other heterogeneous stocks (HSs) that are descended from laboratory strains and from the Diversity Outbred (DO) whose founders include wild-derived strains (Fig. 1a)^11^. The NJ tree for the 54 inbred strains showed the genetic relationships of the strains to the three subspecies: *M. m. domesticus*, *M. m. musculus*, and *M. m. castaneus*. Six strains (A/J, AKR, BALB/c, C3H, C57BL/6, and DBA/2 J) that were founders of both the Boulder heterogeneous stock (BHS)^12, 13^ and the Northport heterogeneous stock (NHS)^13, 14^, and the remaining two founders of these stocks (Is/Bi and RIII for BHS, and CBA/J and LP/J for NHS) were all derived from the same subspecies, *M. m. domesticus*; these two HSs are thus genetically very similar. In contrast, the founder strains of the WHS and DO originated from three subspecies, *M. m. domesticus*, *M. m. castaneus*, and *M. m. musculus*, showing a greater frequency of polymorphisms in the 10,594 genome-wide SNPs in the WHS and DO than in the other HSs (Fig. 1b).

### Designing and establishing a novel outbred stock WHS

**Figure 2:**
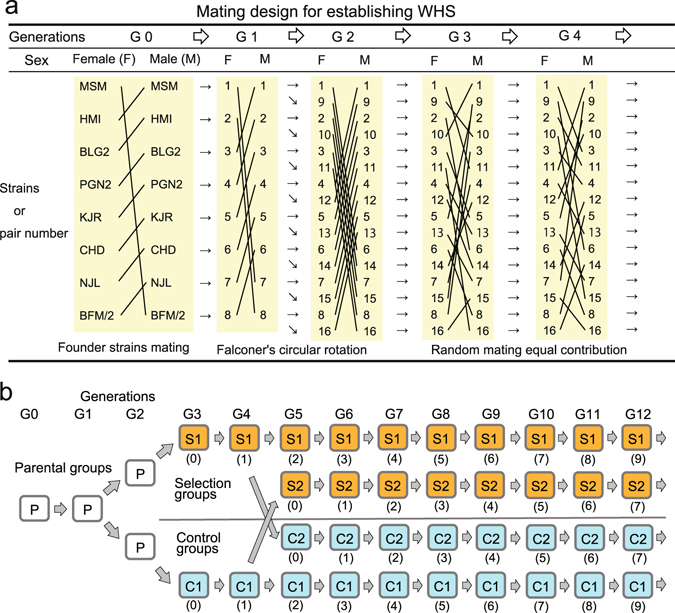
Mating design used to establish the wild-derived heterogeneous stock (WHS) and for selective breeding. (**a**) Mating scheme for the early stage of establishing the WHS. Lines between founder strains and numbers indicate matings to produce progeny. The cage numbers for the next generations are given for the female lineage, so both male and female progeny were given the same cage numbers as their mothers, except for in generation G_1_, in which the number for each female founder strain is given. At generation zero (G_0_), a male mouse of one of the eight wild strains was mated with a female of another wild strain to produce G_1_ mice following the circular rotation rule (see Methods). For example, a female from the MSM strain (no.1) was mated with a male from the BFM/2 strain (no. 8). A male G_1_ mouse from one pair was mated with a female G_1_ from another pair following the circular rotation rule, so a female produced by no. 1 was mated with a male produced by no. 7, a female produced by no. 2 was mated with a male produced by no. 8, etc. At the G_2_ generation, the number of pairs was expanded from eight to 16, and the mice were mated again following the rotation rule. From the G_3_ generation, mating pairs were made following the random mating rule while avoiding intercrossing. (**b**) The process used to establish the two selection groups S1 and S2 and the two control groups C1 and C2. Each number in parentheses indicates the number of selections (see Methods). The orange and blue boxes indicate selection and control groups, respectively.

The new outbred stock WHS was established by rotationally crossing eight founder strains followed by random crossing (Fig. 2a). Choosing an appropriately sized breeding stock is key to preventing the random loss of alleles from the stock because more genetic drift is expected to occur in smaller populations^15^. A large number of breeding pairs should therefore be used to retain as many alleles as possible. However, breeding using a large HS requires large amounts of space and involves a great deal of labor. To determine appropriate experimental design, we simulated the distribution of allele frequencies and the number of lost alleles for a HS descended from 16 breeding pairs under the assumption of no selection for 100,000 realizations of a simple mating scheme. The results of simulation showed that nearly 60% of the genetic loci had lost one or more alleles from the population after 12 generations but that no loci became fixed (Fig. S1). This estimate value was much higher than was previously found for a HS descended from eight breeding pairs, in which 98.2% of genetic loci had lost one or more alleles^15^. We therefore concluded that useful samples for measuring changes in a HS population could be obtained by maintaining the HS using 16 breeding pairs for 12 generations.

### Selective breeding for active tameness using WHS

To clarify the genetic basis of active tameness, we conducted selective breeding for an index of contacting as well as for a secondary selection index, heading, measured using the active tame test (see Methods). Selective breeding was initiated at the G_3_ generation in one group of mice, called S1, and a control group called C1 was not selectively bred (Fig. 2b). Another selection group, called S2, was split from the C1 group and selection was initiated at the G_5_ generation. In addition, another non-selection control group, C2, was split from the S1 group at G_5_ after selection for two generations, and kept without any selection thereafter (Fig. 2b). Splitting into a further two groups was conducted for the following reasons. First, the analyses to detect a selected signature in the genome might be affected by genetic drift. Therefore, to distinguish a selected region from the potentially obscuring factor of genetic drift, we attempted to replicate the results by duplicating the groups. Second, this increased the chance of detecting selected loci, given that each group consisted of a relatively small number of mice.

Up to 80 females and 80 males from each generation and each group were subjected to active tame tests. Since there were no significant behavioral sex differences within all four groups at G_12_ (Wilcoxon test, *P* > 0.05), data for females and males were combined.

**Figure 3:**
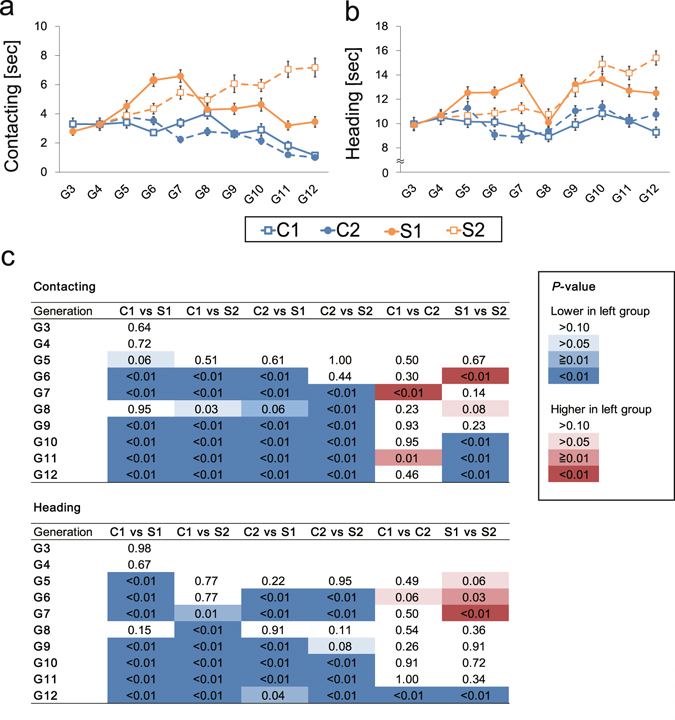
Results of selective breeding for active tameness using the wild-derived heterogeneous stock (WHS). (**a**) The fluctuations in the main selected behavioral trait “contacting” through the generations. The orange and blue lines indicate the mean scores of contacting in selection and control groups, respectively. The dashed lines indicate the split groups. Each dot represents mean score of contacting (n < 160 mice). The error bars indicate the standard errors around the mean (SEM). (**b**) The fluctuations in the second selected behavioral trait “heading” through the generations. The orange and blue lines indicate the mean scores of heading in selection and control groups, respectively. Each dot represents mean score of heading (n < 160 mice). The error bars indicate SEM. (**c**) The results of Steel–Dwass tests between two populations (generation G_3_–G_4_) and four populations (generations G_5_–G_12_).

The transitions found in the behavioral indices contacting and heading through 10 generations are shown in Fig. 3a and b, respectively. Supplemental movies show typical examples of a mouse in selected group S2 (Movie S1) and a mouse in control group C1 (Movie S2). Regarding contacting in the active tame test, the Steel–Dwass test, a non-parametric multiple comparison test, showed that the behavioral indices exhibited significant differences for all four pairs of control and selection groups (C1 versus S1 after G_9_, C1 versus S2 after G_9_, C2 versus S1 after G_9_ and C2 versus S2 after G_7_ (*P* < 0.001; Fig. 3c). For the heading in active tame test, significant differences between types of group were found in three pairs (C1 vs S1, C1 vs S2 and C2 vs S2) but not for the C2 vs S1 pair. For contacting, there were approximately three- and sixfold differences among the mean scores of S1 (3.44 ± 0.35 (SEM)) and S2 (7.17 ± 0.64), and the two control groups (1.14 ± 0.14 and 0.99 ± 0.13 for C1 and C2, respectively). Unexpectedly, the contacting durations for the two control groups decreased as the experiment proceeded (*P* < 0.001 for C1 at G_3_ versus G_12_, and for C2 at G_5_ versus G_12_). This decrease in contacting in the control groups made differences between the selected groups and control groups more pronounced as the experiment proceeded.

The response to selection per generation, *G*, of contacting in both selection groups toward the control (C1) group was positive, and *G* was higher for group S2 (*G* = 2.86) than for group S1 (*G* = 1.48), suggesting that the selective breeding of S2 was more successful than that of S1 even though both selection groups showed positive selection indexes. The realized heritability (*h* ^2^) was estimated using the average contacting and heading values for generations G_5_–G_12_ for group S2 and for generations G_3_–G_12_ for group S1. The estimated *h* ^2^ values for contacting in groups S2 and S1 were 0.205 ± 0.1 (SEM) and 0.07 ± 0.045, respectively, suggesting that contacting is heritable, albeit with low heritability in mice, consistent with a previous study^9^. The estimated *h* ^2^ values for heading in groups S2 and S1 were 0.393 ± 0.421 and 0.194 ± 0.206, respectively. The high standard errors might be because heading was used as a secondary selection index and rarely used for selecting the mice for breeding.

### Selection mapping of genetic loci for contacting

**Figure 4:**
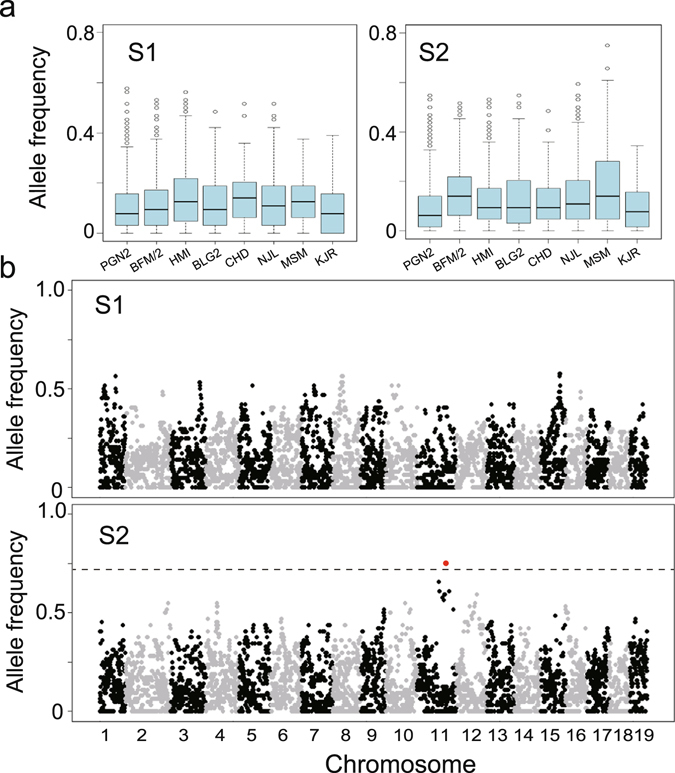
Contributions of one-strain-specific single nucleotide polymorphisms (SNPs) in the wild-derived heterogeneous stock (WHS) and the selection mapping results. (**a**) Genome-wide average frequencies for 20,530 one-strain-specific SNPs in groups S1 and S2 in generation G_12_ are indicated. (**b**) Selection mapping results for groups S1 and S2. For group S1, no SNP reached the threshold determined using the computer simulation described in Table S2. For the S2 group, one SNP (UNC20197962) marked with a red dot exceeded the threshold value for the MSM strain (a dashed line).

To identify genetic loci associated with active tameness, we conducted genome-wide SNP analyses of the selected WHS populations using the GigaMUGA array, which covers 144 K SNPs^16^, greater than that of MegaMUGA array. In the genotypes of the eight founder strains of the WHS, those SNPs private to a single founder were mostly in the two *M. m. domesticus* strains, PGN2 (n = 8,109 SNPs) and BFM/2 (n = 6,038 SNPs), followed by the *M. m. castaneus* strain HMI (n = 4,533 SNPs). The five *M. m. musculus* strains had fewer private SNPs (Table 1). Given that the GigaMUGA array was mainly optimized for the analysis of *M. m. domesticus* mice, fewer SNPs for distinguishing *M. m. musculus* strains are included, so these statistics may be subject to ascertainment bias. The genomic contribution of the founder strains to each group of WHS mice at the G_12_ generation was estimated using private SNPs alone. The average frequencies of private SNPs for the eight founder strains in the 32 mice used to breed the next generation of each of the four groups S1, S2, C1, and C2 at generation G_12_ were calculated, and the results are shown in Figs 4a and S2a. The allele frequency for each strain was expected to be 0.125 because of the strain-specific (private) SNPs that were used. We found a mean allele frequency for strain-specific SNPs for each founder of close to 0.125 for each group (0.126 ± 0.020 (mean ± standard deviation), 0.128 ± 0.016, 0.122 ± 0.017, and 0.126 ± 0.026 for groups C1, C2, S1, and S2, respectively).

**Table 1:** Strain differences in single nucleotide polymorphisms (SNPs) from the GigaMUGA array.

Position	Strain	Subspecies group	# of SNPs	%
1 strain specific	PGN2	*M.m.domesticus*	8,109	15.6
	BFM/2	*M.m.domesticus*	6,038	11.6
	HMI	*M.m.castaneus*	4,533	8.7
	NJL	*M.m.musculus*	724	1.4
	BLG2	*M.m.musculus*	585	1.1
	MSM	*M.m.musculus*	275	0.5
	CHD	*M.m.musculus*	158	0.3
	KJR	*M.m.musculus*	108	0.2
Sub-total			20,530	39.4
2 strain identical			19,719	37.8
3 strain identical			9,167	17.6
4 strain identical			2,719	5.2
Total			52,135	100

“1 strain specific” is SNPs private to a single founder strain. “%” was calculated as follows. (# of SNPs / Total) * 100.

In the two selected groups S1 and S2, a significant increase in the behavioral score for contacting was observed at G_12_ (Fig. 3). To identify loci associated with increased contacting, we conducted selection mapping using the SNP data from the two selected groups, S1 and S2 (Fig. 4b). In the mapping, we examined whether any allele frequencies increased more than would be expected from random genetic drift. We compared the actual allele frequency for each private SNP with the simulated allele frequency based on random genetic drift (i.e. the non-selection model) to identify these loci. We calculated genome-wide thresholds (*P* < 0.05, Bonferroni correction) for the maximum allele frequencies using a computer simulation based on a non-selection model (see Methods). The genome-wide thresholds for significant increases in allele frequencies more than expected through random genetic drift for the different strains within the groups were 0.703–0.797 (Table S2). In the selected group S2, the observed allele frequency of SNP UNC20197962 (96,688,668) on chromosome 11, which was private to the MSM strain, reached 0.75 (maximum allele frequency in the simulation based test: *P* = 0.0017), which exceeded the threshold for the MSM strain (Table S2). The other selected group, S1 (Fig. 4b), and the control groups C1 and C2 (Fig. S2b), had no SNPs for which the frequency significantly increased (Table S2). We found that the allele private to MSM for UNC20197962 was lost in the two groups S1 and C2, but remained at a low frequency (0.188) in C1. These data suggest that the MSM-derived allele of UNC20197962 had been lost due to genetic drift during the early stage of breeding before C2 was separated from S1 (earlier than G_4_). This also explains why the S1 group did not exhibit selection at this SNP.

**Figure 5:**
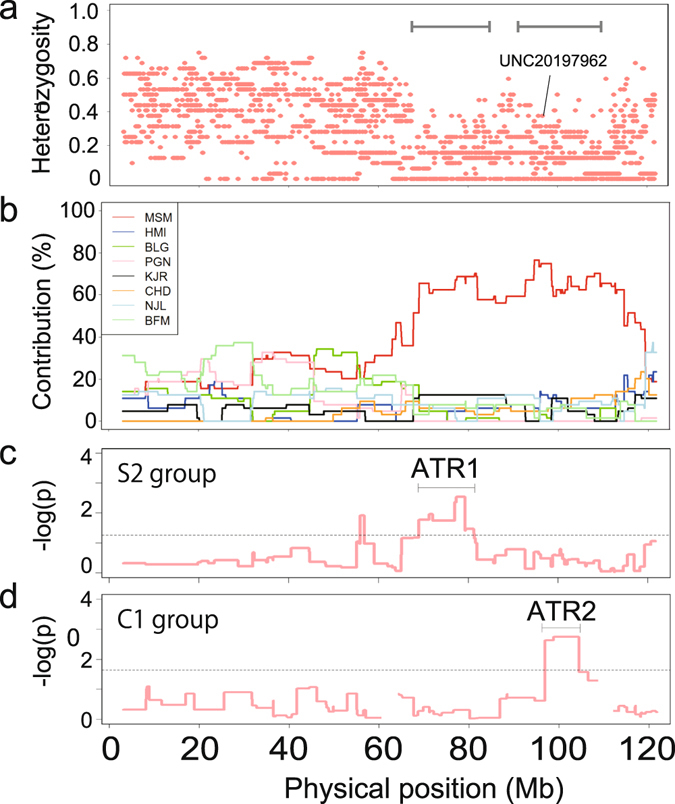
The haplotype derived from the MSM strain was selected in group S2 in generation G_12_. (**a**) Heterozygosity determined using overall genotyped single nucleotide polymorphism (SNP). A solid line indicates the SNP (UNC20197962) exceeded the threshold value for the MSM strain. The solid horizontal line indicates the region showing decrease of the heterozygosity. (**b**) Contributions of inferred haplotypes from the eight founder strains. (**c**) Results of linear mixed model association analysis of contacting and the inferred MSM haplotype using group S2. (**d**) Results of Cox mixed model association analysis for group C1.

We analyzed heterozygosity and found a loss of heterozygosity around the UNC20197962 SNP region in the S2 group (Fig. 5a) but no loss of heterozygosity in the C1 group (Fig. S3). The region around the UNC20197962 SNP in group S2 exhibited loss of heterozygosity at the genome-wide level (Fig. S4). These results suggest that selection in this region of the genome occurred in group S2. To increase the resolution to detect genomic regions under selection by haplotype analysis, we inferred the founder haplotype from a random forest^17^. The major contribution to the inferred haplotype in this region is from the MSM strain (Fig. 5b). The haplotype block of MSM including UNC20197962 is approximately 52 Mb, and lies between 67.7 and 119.7 Mb on chromosome 11.

### Fine mapping using association analysis

To fine-map genetic loci associated with contacting within the 52 Mb haplotype block, we conducted association analysis on chromosome 11 between behavioral traits and the inferred haplotype of MSM. If the MSM allele in the candidate region increases contacting during selective breeding, an association study using selection group S2 should show a significant association of the 52 Mb region with contacting (Fig. 5c). In addition, control group C1, which retained the UNC20197962 allele private to the MSM strain at a frequency of 0.188, could have contained genetic information for increased contacting at a lower level (e.g., giving a duration of 0–1). We therefore used data for the S2 and C1 groups at generation G_12_. Because the phenotypic distributions for contacting were very different between S2 and C1, we applied different association analyses to each group. Contacting in the S2 group followed an approximately Normal distribution (Shapiro-Wilk test, *P* > 0.05), for which we used a linear mixed model. In the 52 Mb haplotype block, a 12.2 Mb region between 69,042,047 and 81,256,559, was significantly associated with contacting (Fig. 5c), and the MSM haplotype showing higher contribution than the other haplotypes (Fig. 5b). We called this locus active tameness region 1 (ATR1). Many mice in group C1 gave zero scores for contacting because group C1 was not selectively bred, and therefore could not be transformed to a Normal distribution. We therefore modeled the contacting phenotype for group C1 as an uncensored latency measure (the time until contacting ceased). Using a mixed effects Cox model, we found another locus, which we called active tameness region 2 (ATR2), that was significantly associated with contacting. ATR2 was approximately 7.5 Mb wide, and was found at 96,888,139–104,407,876, on the distal side of ATR1 within the haplotype block (Fig. 5d).

Tameness is potentially associated with levels of anxiety and fear toward humans or the human-mediated environment^1, 3^. Therefore, genes related to behaviors such as anxiety, fear, exploration, novelty seeking, and social behavior could be associated with tameness. We searched genes within the ATR1 and ATR2 regions for these nine key words in the Mouse Genomics Informatics database and extracted 29 out of 569 protein coding genes. Among these extracted genes, 22 were found to be expressed in the mouse brain (Table 2). These results suggested that one or more of these genes might be associated with active tameness in mice.

**Table 2:** Profiles of 28 genes within ATR1 and ATR2 using keywords related to tameness.

ATRs	Start	End	Symbol	Keywords	Number of keywords	Expression	
						Enbryonic head	Postnatal brain
ATR1	69632990	69653297	*Fxr2*	anxiety, fear	2	+	+
	69823122	69837784	*Nlgn2*	anxiety, fear, novelty, social, thigmotaxis	5	ND	ND
	70017085	70045532	*Dlg4*	anxiety, approach, exploration, fear	4	+	+
	70614883	70619216	*Chrne*	exploratory	1	ND	ND
	71749920	71789647	*Wscd1*	exploration, novelty	2	+	ND
	73019008	73042073	*Camkk1*	fear	1	+	+
	73160421	73172685	*P2rx5*	exploration, exploratory, fear, novelty	4	+	ND
	73183596	73199042	*Ctns*	anxiety, fear, thigmotaxis	3	ND	ND
	73234292	73261242	*Trpv1*	anxiety, exploration, fear	3	+	+
	73304992	73329596	*Aspa*	anxiety, fear	2	+	+
	74906509	74925948	*Srr*	anxiety, fear, social, thigmotaxis	4	+	+
	76998603	77032340	*Slc6a4*	anxiety, approach, exploration, fear, novelty, social	6	+	+
	77493562	77507786	*Git1*	anxiety, fear	2	ND	ND
	78166106	78176675	*Nek8*	social	1	ND	ND
	79013440	79146407	*Ksr1*	fear	1	+	+
	79339693	79581612	*Nf1*	exploratory, fear	2	+	+
	80477023	80481184	*Cdk5r1*	anxiety, fear, exploration	3	+	+
ATR2	97205842	97280638	*Npepps*	anxiety, exploration, fear, social, thigmotaxis	5	+	ND
	97509340	97576186	*Srcin1*	exploration	1	ND	ND
	98740638	98769006	*Thra*	anxiety, fear, exploratory, social	4	+	+
	100761069	100762931	*Hcrt*	social	1	+	+
	101070012	101077672	*Naglu*	anxiety, fear	2	−	ND
	101078411	101080527	*Hsd17b1*	exploration, novelty	2	ND	ND
	102145513	102149477	*Nags*	social	1	−	ND
	102430315	102437048	*Grn*	aggression, anxiety, fear, exploration, novelty, social, thigmotaxis	7	+	+
	104132855	104175523	*Crhr1*	anxiety, exploration, exploratory, fear, social	5	+	ND
	104231390	104332090	*Mapt*	anxiety, approach, fear	3	+	+

ND, no data was registered. “−”, expression was not detected. “+”, expression was registered in the Gene Expression Database. The gene information was obtained from Gene Expression Database in Mouse Genomics Informatics.

### Comparative analysis with dogs

**Figure 6:**
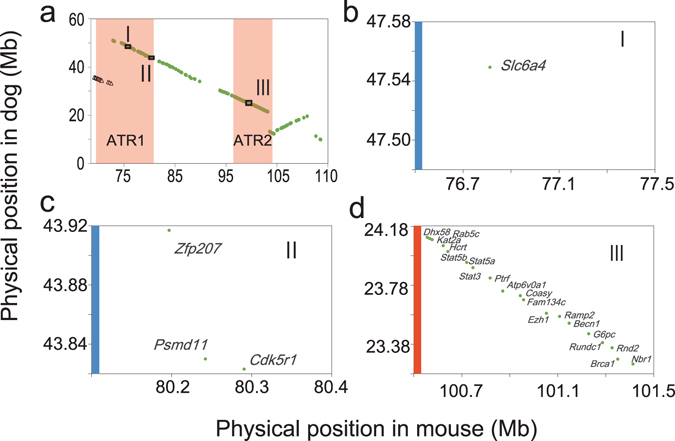
Comparative analysis of the mouse and dog genomes. We performed a comparative analysis of the mouse and dog genomes to determine whether the syntenic region of ATR1 was selected during the domestication of dogs. We first downloaded gene data for orthologues from Ensembl (Archive EnsEMBL release 67; NCBI m37 for mouse and CanFam2.0 for dog). We then used selective sweep datasets that referred to the domesticated region from previous publications18,19. Overlapping genes between the orthologues and the selective sweep regions were then extracted. (**a**) Plot of the genes on mouse chromosome 11 expressed in mouse brain (x-axis) against synteny in dogs (y-axis). Triangles are dog chromosome 5, circles are dog chromosome 9. (**b**–**d**) Syntenic regions of mouse chromosome 11 in dogs and regions that have been selected during the domestication of dogs, identified by performing genomic comparisons of grey wolves and dogs (vertical blue line) and of 10 domesticated dog breeds (vertical orange line).

If the selected genes for contacting are also associated with active tameness in mice, the orthologs in other species might also be associated with tameness and/or domestication. If so, these genes should have been selected during the domestication of those species. Given that dogs have been intensely domesticated and exhibit high levels of tameness^18^ and that there are genomic analyses for selected regions during domestication^19, 20^, it is possible that there is a signature of selection for tameness in the dog genome. To investigate whether the syntenic regions of ATR1 and ATR2 have also been selected during dog domestication, we conducted a comparative analysis of the genomes of mice and dogs. In previous studies, genomic regions in which a selection had occurred were reported using genomic comparisons between gray wolves and Chinese indigenous dogs^20^, and among 10 different dog breeds^19^. We compared ATR1 and ATR2 in the mouse genome with syntenic regions in the dog genome, and found that the syntenic region contains three selective sweep regions in the dog genome which were identified previously^19, 20^ (Fig. 6a). To examine whether these overlaps are likely to have occurred by chance, we estimated the probability of overlap of ATR1 and ATR2 with randomly selected selective sweep regions in the dog genome and found that the overlap was unlikely by chance (*P* = 0.051 for ATR1 and *P* = 0.096 for ATR2). Therefore the overlap of the ATR1 and ATR2 with the selective sweep regions in dogs is probably not coincidental, suggesting these regions could play similar roles in both mice and dogs. The three syntenic regions for mice included 23 genes expressed in the mouse brain (Fig. 6b–d and Table S3). Among these 23 genes, three (solute carrier family 6, member 4 (*Slc6a4*); cyclin-dependent kinase 5, regulatory subunit 1 (*Cdk5r1*); and hypocretin neuropeptide precursor (*Hcrt*)) overlapped with the 28 genes extracted by performing a keyword search in the ATR1 and ATR2 regions (Table 2). These genes could be associated with tameness in mice and domestication in dogs.

## Discussion

In the present study, we established a novel resource, WHS mice, from eight wild strains. We successfully induced changes in tameness behavior after selective breeding using genetically heterogeneous WHS mice. In the comparison of genomic SNPs between selected and control groups, and using a simulation data based on a non-selection model, we found a genomic signature of selection on chromosome 11 at two closely located loci.

The contacting durations in both control groups decreased as the generation number increased in the selective breeding experiment. It is difficult to explain why this happened without any deliberate selective breeding. One possibility is that contacting decreased because of genetic drift in the control population as the generation number increased. However, this is less likely because the contacting scores for both control groups changed in a similar way. A second possibility is that unintentional selection occurred in the control groups. However, we selected mice to form breeding pairs randomly in each generation and carefully attempted to avoid unintentional selection occurring in the control groups. Also, there was no difference in the management or handling of the mice between selected and control groups throughout the breeding experiments. A third possibility is that the control group phenotypes changed because of the accumulation of genetic recombination, resulting in the genomic structure changing, particularly in the early WHS breeding phase. Selective breeding of the HS started at the third generation of crosses using eight inbred strains. This was different from the selective breeding procedures used in other studies, selection having been started in later generations in those studies. Our method is useful because it allows the genomic allele frequencies to be calculated precisely from the breeding scheme. However, the haplotype was very large in the early breeding phase and gradually became shorter as the generation number increased. The change in haplotype length dramatically increased genetic diversity in the population but may also have affected the phenotype. It will be interesting to examine in future work how changes in haplotype length affect the phenotype as the generation number increases.

The contacting scores for different generations of the selective breeding groups were very different. In particular, the S1 contacting scores were significantly different from the control group contacting scores, but the contacting scores were low. The fluctuations in the contacting scores were similar to the fluctuations found in an early study of selective breeding in an open-field setting^21^. An apparent response to selection was observed in the average score for each selected group, but there was considerable inter-generation variability. We think that the differences between the contacting scores of the selected and control groups will become more apparent in later generations because this was found in a later study, also performed by the authors^22^.

We identified a selected region based on allele frequencies that exceeded a threshold determined by the simulation for allele frequencies using pedigree and founder genotype data. Recent genomic studies in which selection mapping was conducted for related domesticated and wild animals revealed genetic loci associated with animal domestication^7, 8^. The WHS will be more useful than comparisons of domesticated and wild animals for selection mapping of complex traits. The primary reason for this is that there are advantages to using pedigree information and genomic data on founder strains in the case of WHS, while these data are neither available for wild animals nor for most domesticated ones. The availability of the pedigree and genomic data for the founder strains allows us to perform accurate fitting using the rules of genetic inheritance. The second reason is that the environmental conditions of the mice are controlled better for WHS than for other wild and domesticated animals. Given that environmental variance affects the phenotypic variance of the traits^23^, the circumstances in the laboratory, with the animals being kept in a highly similar environment, allowed us to minimize environmental effects. For this reason, the proportion of genetic variance relative to the phenotypic variance of the trait in the laboratory animals is probably larger than that among non-laboratory populations. Based on the highly controlled environmental conditions for laboratory mice, selection mapping using WHS is a powerful method for revealing the genetic regions associated with complex traits.

We found significant associations of contacting with two loci, ATR1 and ATR2. These two loci were included in the 52 Mb haplotype region from MSM with higher allele frequencies. The MSM strain did not exhibit high levels of active tameness, but it is possible that interactions between ATR1 and ATR2 and other loci with alleles from other strains could have increased active tameness in group S2. The existence of two closely located loci could be one of the reasons why the selected region shows a large haplotype block. ATR1 and ATR2 overlapped with the two sub-regions where large reductions of heterozygosity were observed. Interestingly, in the association study, we found a locus associated with active tameness in ATR2 in the control group C1 but not in group S2. We assume that ATR2 played a role in increasing active tameness during the early selective breeding phase but that ATR1 had a stronger effect in the later stages in fixing active tameness within the group. We could not identify clearly how the effects of the two loci were different. The genomic profiles of other minor loci would have changed during selective breeding even though the differences were not detectable, so the effects of ATR1 and ATR2 may have changed because of different epistatic interactions between multiple loci.

Active tameness is a complex trait, so associations between multiple genetic loci and contacting can be expected. However, through our genetic analysis, we detected associations only between ATR1 and ATR2 on chromosome 11 and tameness. One limitation of our analyses could have been caused by each locus for contacting having a small effect. We expected that the threshold set from the allele frequency simulation for each generation performed using the non-selection model would have eliminated many loci that had weak selective pressures, such as relatively small frequency changes. In such cases it could be difficult to detect genetic loci with small effects. Paradoxically, the MSM haplotype of the ATR regions was lost in the S1 group but the S1 group responded to selection for active tameness, suggesting that other loci must be involved in contacting behavior. Another limitation of the study was related to ascertainment bias caused by the SNP being identified using the GigaMUGA array. Between 108 and 724 strain-specific SNPs were identified in the five strains derived from *M. m. musculus*, whereas many more strain-specific SNPs (4,533–8,109) were found for the other strains. This meant that the ability of the method to detect loci related to active tameness was limited. We expect the ability of the method to detect loci related to active tameness to be improved if more strain-specific SNPs for *M. m. musculus* can be identified.

The dog database analyses allow us to identify three genes (*Slc6a4*, *Cdk5r1*, and *Hcrt*) as candidate genes associated with active tameness in mice. One of the candidate genes in ATR1 was *Slc6a4*, an integral membrane protein that transports the neurotransmitter serotonin and is involved in the pharmacological targeting of psychomotor stimulants such as amphetamine and cocaine. It has been reported that *Slc6a4* is associated with aggressive behavior^24^, social behavior^25^, and anxiety-related responses in mice^26^. Interestingly, selection mapping using genomic data for dogs and wolves has shown that *SLC6A4* was selected during the domestication of dogs^20^. Notably, the downstream metabolite of a transport substrate of *SLC6A4* was found to be associated with aggressive behavior in dogs in another association study^27^. The other leading candidate gene, located in ATR2, was *Hcrt*, which encodes a hypothalamic neuropeptide precursor protein giving rise to orexin. Orexin has been shown to affect sleep-related phenotypes in mice^28^ and dogs^29^. Orexin knockout mice have been found to exhibit diminished behavioral responses to stress in resident–intruder test, suggesting that *Hcrt* products may be associated with the “fight or flight” response^30^. It is therefore possible that *Slc6a4* and *Hcrt* play important roles in changing the traits of active tameness during selective breeding in mice.

In conclusion, we found that two adjacent but distinct genomic loci ATR1 and ATR2 were associated with tameness in mice. However, we could not identify the gene(s) that cause active tameness, and other regions could also be associated with active tameness. Further selective breeding and control group breeding will allow recombination events to accumulate, resulting in a shorter haplotype block in later generations. Genetic analyses of samples from later generations, obtaining better resolution, outcrossing between selected and control groups to give phenotypic diversity, and performing quantitative trait locus analysis (e.g., genome-wide association studies) could allow the gene(s) that cause active tameness to be identified. It would also be useful to perform transcriptome analyses to allow multiple genes related to active tameness in mice to be identified.

## Methods

### Animals

All mice were bred and kept under specific-pathogen-free conditions at the National Institute of Genetics (NIG) in Japan. Food and water was available *ad libitum*, and a 12 h light and 12 h dark cycle was used. The mice were housed in a temperature-controlled room at 23 ± 2 °C. Eight founder strains were derived from wild mice (Table S1)^31–33^ and kept at the NIG. In the case of cage exchange and behavioral tests, each mouse was gently caught by its tail using tweezers covered with silicon tubing to minimize pain.

Mice were maintained and all experiments were performed in accordance with NIG guidelines and all procedures were carried out with approval (No. 26–9) from the Committee for Animal Care and Use of the NIG.

### Genetic structure of WHS founders

To clarify the genetic characteristics of the WHS, we conducted an analysis of genomic polymorphisms in the eight founder strains. Genomic DNA obtained from the eight strains was genotyped using the 77 K MegaMUGA array (Geneseek, Lincoln, NE, USA), which is an SNP marker genotyping array based on the Illumina platform^10^. To obtain additional data for other mouse strains, we downloaded available SNP data for MegaMUGA for 45 mouse strains including other HS founder strains from UNC Systems Genetics at the University of North Carolina (http://csbio.unc.edu/CCstatus/index.py?run=GeneseekMM). We then used the data for the 54 strains in our analysis (Table S1). Using PLINK v. 1.9^34^, the nucleotide types that were identical among all strains and missing sites were removed, and finally data on 10,598 SNPs were obtained.

The evolutionary histories of the WHS founder strains and other inbred mouse strains were inferred from data acquired by performing NJ using the p-distance, which is the number of different polymorphic nucleotides in two sequences divided by the length of the sequence. This allowed the relationship between the strains to be identified. We used MEGA version 6.06^35^ to construct a NJ tree. Bootstrap tests were conducted 1,000 times.

### Establishing the wild-derived heterogeneous stock

The eight wild strains BFM/2, PGN2, HMI, BLG2, NJL, KJR, CHD, and MSM were mated to establish the WHS (Fig. 2a). At generation zero (G_0_), a male mouse of one of the eight wild strains was mated with a female of another wild strain to produce G_1_ mice following the circular rotation rule^15^ (Fig. 2a). A male G_1_ mouse from one pair was mated with a female G_1_ from another pair following the circular rotation rule. At the G_2_ generation, the number of pairs was expanded from eight to 16, and the mice were mated again following the rotation rule. At the same time, the groups were expanded into two groups, each of which consisted of 16 pairs, so as to avoid losing eight different genomes from the stock. From the G_3_ generation, mating pairs were made following the random mating rule while avoiding intercrossing. Subsequent mating was performed with 16 pairs in each group. In these generations (i.e., before selective breeding), at least one female and one male offspring from each pair was required to form a subsequent pair. If a pair did not produce any progeny even after one month of being paired, the subsequent pair was formed from a littermate or from the next litter produced by the same parents. In cases in which a mating pair did not produce any progeny even after making the next additional pair, the progeny from other pairs within the same group were used as substitute mice, which is also appropriate to avoid inbreeding. As a result, at least 14 breeding pairs produced progeny in each group at each generation during G_3_ to G_12_. At the G_3_ generation, the genomes of all eight strains were mixed randomly in each mouse, so genetic heterogeneity between mice was expanded more extensively than in the eight founder strains. Given this approach, a higher level of phenotypic diversity is expected in the WHS.

### Selective breeding

We conducted breeding for two groups of selection and non-selection (control) from G_3_ (Fig. 2b). Each group was split into a further two groups, selected and non-selected, from G_5_. The first two lines were called selection 1 (S1) and control 1 (C1), and the additional two groups after splitting were selection 2 (S2) and control 2 (C2), which were derived from C1 and S1, respectively.

The selection criteria applied only to the selection groups. The mice used for breeding were chosen based on highest scores of the behavioral indices, contacting and heading toward human hand, in the active tame test among five females and five males. Contacting and heading were defined as the duration of making contact with a human hand and the duration the head was turned towards the hand, respectively, in active tame tests, as described previously^9^. Offspring were ranked for contacting score within five male progenies and five females, which were produced from each pair. When the number of progeny for each sex was less than five in each pair, the mice for next mating were chosen from the smaller number of progenies. When the highest contacting scores were equal between two mice, the mouse that exhibited a higher heading score was chosen for the next mating. Crosses within each family were not used to avoid the effect of inbreeding and environmental variance from the family^36^. Each selected male and female from one family was mated to a selected female and male of another family, respectively, to produce the next generation. For control lines, first, a randomly selected mouse for each five males and five females in each family was chosen and mated with a similarly chosen female and male, respectively, from another family to avoid inbreeding. Consequently, up to 80 mice for each sex were tested for each group at each generation.

### Behavioral assay

In accordance with our previous work^9^, tame tests were conducted using over 400 mice of 6 weeks-of-age for each generation. The tests were implemented during the light period using an open-field apparatus consisting of a gray square measuring 40 × 40 × 40 cm (O’Hara & Co. Ltd., Tokyo, Japan) and illuminated with 100 lux at the center of the field. The active tame test was video-recorded using a digital camera (CX5; Ricoh Company, Ltd., Tokyo, Japan). The duration of events for each trait was measured by human observation at a resolution of 0.1 s using tanaMove software (version 0.01), which is freely available from the website of the NIG (http://www.nig.ac.jp/labs/MGRL/tanaMove.html)^9^. In the active tame tests, each mouse was gently caught by its tail using tweezers covered with silicone tubing, to minimize pain, and gently placed in the center of the field. An experimenter placed his hand, in a plastic glove, at the bottom of the test field and attempted to keep the hand approximately 10 cm from the mouse. The fingers were continually moved slightly during the test so that the mouse realized that the hand was part of a human body. If a mouse moved away, the hand followed it to maintain the required distance. However, if a mouse headed towards or came into contact with the hand, the hand was kept in the same position, with the fingers being moved slightly. The duration a mouse headed towards the hand and the duration the mouse was in contact with the hand were measured by inspecting the relevant movie file. Experimenter T. G. ceased performing the active tame tests and experimenter Y. M. started performing the tests (after being carefully trained) at generation G_8_ to decrease the effect the experimenter had on the results. Observations were made by the same person (not involved in the selective breeding process and not the experimenter performing the behavior tests) for all the tame tests. The meanings of the video-recorded files were not revealed to the observer at any time during the study.

### Estimation of response to selection, *G*, and realized heritability

The qualities of the selective breeding process for the two selection groups were evaluated by estimating the responses to selection via the selection index (contacting) using data for groups C1 and S1 between generations G_3_ and G_12_ and using data for groups C1 and S2 between generations G_5_ and G_12_. The response to selection, *G*,  was defined as the difference between the mean for the selected group (S2) and the mean for the control (C1) group, and the mean responses were calculated.

Heritability, *h* ^2^, and its standard error were estimated using the realized heritability of contacting and heading calculated using a previously described method^36–38^. The realized heritability was estimated by performing regression analyses on the relationships between the responses to selection and the cumulative selection differentials between generation G_3_ (for group S1) or G_5_ (for group S2) and generation G_12_^37^. The selection differentials within a family and for one sex were defined as the differences between the means for selected mice and the means for their offspring. These values were averaged within a line^36^. The response to selection was calculated as described above. The standard errors of realized heritability were calculated by dividing the sampling variance of the response by the cumulative selection differentials^38^. The variance was obtained by using the data of estimated *h* ^2^ and C1 group as a control.

### Haplotype inference of founder strains

To clarify the genetic contribution of the founder strains to each WHS mouse, we inferred the haplotypes for each local genomic region in the autosomes. In this analysis, 32 WHS mice from each of C2, S1, and S2, and 128 mice from C1 at G_12_ and eight founder strains were genotyped using the 144 K GigaMUGA SNP array (Geneseek)^16^. Using PLINK v. 1.9^34^, we removed SNPs with identical nucleotides among the eight founder strains and those for which 1% or more of the data were missing. In addition, the SNPs located on the X and Y chromosomes were also removed because these chromosomes have different recombination rates from the autosomes, which would affect the results of subsequent analyses. Finally, we obtained data on 52,135 SNPs of 128 WHS mice and eight founder strains (Table 2).

To infer the haplotypes of WHS founder strains, we used Beagle v. 4.0^39^ and RFMix v. 1.5.4, a program for local-ancestry inference that uses a discriminative approach with a conditional random field parameterized by random forest. The program was trained to distinguish between founder strains using the reference genotype for each founder strain^17^.

### Identifying the selected region

By conducting selective breeding, the allele frequency for particular SNPs associated with tameness would increase through the generations. Individual SNPs should be passed from parents to offspring in a pedigree according to the law of inheritance. The frequency of an SNP in an arbitrary generation in our study could be simulated under a no-selection model. Then, we conducted a statistical test to detect an increase in allele frequency. Given that the number of SNPs is large, we encountered the multiple testing problem. Although we can adjust for this problem using standard methods, the threshold becomes smaller and the power falls slightly as the number of SNPs increases. Importantly, many SNPs are linked on the same chromosome and are transmitted together in a pedigree. To resolve these problems, a statistical test for an increase of SNP frequency by whole-genome simulation was established as follows: (1) The SNPs that are strain-specific alleles on 19 autosomes were used. (2) The pedigree realized in the experiment was used for the simulation. (3) The nucleotide types of each SNP in eight strains were determined by GigaMUGA. (4) SNPs were allocated to positions in the genome according to their genetic distances obtained from the Mouse diversity array^40^. Using the above settings, one simulation run used the following assumptions: (1) Prior to each fertilization, recombination occurred at random in the genome. (2) After recombination, the 19 autosomal chromosomes were independently inherited by the offspring. (3) No artificial/natural selection acted on the SNPs. (4) At G_12_, the maximum frequency of all SNPs was recorded. This process was repeated 10,000 times. The recorded frequencies were used as the simulated null distribution. The rejection region (genome-wide significance level) was set at the 5th percentile of the distribution. We considered any region near the SNPs that exceed the threshold as a candidate region for tameness. The above simulation was conducted using one strain unique 20,530 SNP for two selected populations (S1 and S2). Due to the large number of simulations for each founder strain (8 times) and for each group (2 selected groups), we used the Bonferroni correction to account for multiple comparison. Furthermore, additional analyses were performed using two control groups (C1 and C2) to evaluate our simulation method.

The frequency of every heterozygosity observed in a particular genotype was used to determine the observed heterozygosity *H*_*O*_, which is an index used to quantify genetic diversity and to identify a selected region. Overall 51,649 SNPs of S2 and C1 group were used in the analyses. The 32 mice of each groups were subjected to the calculation of heterozygosity for each SNP. A region could be selected if heterozygosity was lost in that region relative to other regions. We used a sliding window of 100 SNPs with a 10 SNP jump, and detected the lowest 10% of the windows genome-wide as candidate selected regions.

The SNP data for the WHS founders were available, enabling us to determine which strain was selected in a particular region. The inferred haplotype data described above were used to predict which strains would be selected.

### Association analysis on tameness

Given that the selected region was observed for the MSM haplotype in the posterior part of Chromosome 11, the results suggesting that the haplotype has the effect of increasing the contacting. To examine the association between the MSM haplotype based on inferred haplotype data (described above) and contacting, we used a mixed linear model for the S2 group (n = 32). The C1 group also had MSM SNPs in the selected region, but the genetic basis for low levels of contacting by mice in C1 and S2 groups were potentially different. Therefore, we also performed association analysis on the C1 group data. For the C1 group (n = 128), due to the presence of zero scores for contacting, the mixed effects Cox model was used. Inferred haplotype data (described above) were used in this analysis. The analyses were performed using 4,198 SNPs (S2 group) and 3,943 SNPs (C1 group) on mouse chromosome 11 using the inferred haplotypes derived from the MSM strain or the seven other wild strains. The number of MSM haplotype on each SNP locus for Chromosome 11 and contacting were used as explanatory and response variables, respectively. Family data were used as random effects. The likelihood ratio test was used for each analysis on the SNP locus. A false discovery rate (FDR) was applied to correct for the multiple comparison (q = 0.10). These analyses were undertaken using the R version 3.0.2 and additional R packages, lme4 version 1.1.7, coxme version 2.2.4 and qvalue version 1.35.0.

### Search for candidate genes and analysis of syntenic regions

To identify the candidate genes and to obtain further information, we searched the Mouse Genome Informatics database (MGI 6.07; accessed on February 22, 2017) for the genetic region that reached the genome-wide significance level. Because we focused on contacting, which is associated with active tameness, the genes should be expressed in mouse brains and associated with tame-related behavior. Therefore, we used a keyword search in MGI for ATR1 and ATR2 (69,042,047–81,256,559 and 96,888,139–104,407,876 on chromosome 11, respectively) using the terms aggression, approach, anxiety, exploration, exploratory, fear, novelty, thigmotaxis and social as tame-related behaviors. Next we searched for genes expressed in the brain for the ATR1 and ATR2 region using the Gene Expression Database (GXD) in MGI. We cited the genes exhibiting expression in the head of embryos or the brains of adult mice.

We determined whether the syntenic region of ATR1 and ATR2 was selected during dog domestication by performing comparative analyses of the mouse and dog genomes. We first downloaded gene data for orthologues from Ensembl (Archive EnsEMBL release 67; NCBI m37 for mouse and CanFam2.0 for dog). We then used selection datasets from previous publications^19, 20^, that referred to the domesticated region. Overlapping genes between the orthologous genes and the genes in the selected region were then extracted.

We used the simulation method to estimate the probability that the selected region in mice and the selective sweep region in dogs overlapped. We used a total of 354 previously described segments as selective sweep regions^19, 20^. The orthologous genes in mice and dogs were downloaded from Archive EnsEMBL release 67. In the simulation, randomized regions in the mouse genome (ATR1 (12.21 Mb) and ATR2 (7.51 Mb)) were collected and the gene set was first prepared. The positions in orthologous dog genes corresponding to the extracted mouse genes were then extracted. The number of selective sweep regions overlapped with the syntenic region for dogs was then determined using the orthologous gene dataset. The probabilities of observing more than two or one overlaps with regions of the same lengths as ATR1 (12.21 Mb) and ATR2 (7.51 Mb), respectively, were estimated across the 10,000 samples.

## Electronic supplementary material

Movie S1

Movie S2

Supplementary information
