# Supplementary material for: Selective breeding and selection mapping using a novel wild-derived heterogeneous stock of mice revealed two closely-linked loci for tameness
Source: Sci Rep. 2017 Jul 4;7:4607. doi: 10.1038/s41598-017-04869-1 (PMC5496859; doi:10.1038/s41598-017-04869-1)
Supplement: Supplementary file 3 — Supplementary information [file 41598_2017_4869_MOESM3_ESM.pdf]

Selective breeding and selection mapping using a novel wild-derived heterogeneous stock of mice revealed two closely-linked loci for tameness

Yuki Matsumoto, Tatsuhiko Goto, Jo Nishino, Hirofumi Nakaoka, Akira Tanave, Toshiyuki Takano-Shimizu, Richard F Mott, Tsuyoshi Koide

Supplementary information

Table S1. Origins of the WHS founder strains and the 54 strains used in the phylogenic analysis.

Table S2. Threshold for each strain-specific SNP after Bonferroni correction and the observed maximum allele frequencies for the eight founder strains in the four WHS groups.

Table S3. Profiles of 23 mouse genes in the syntenic region of the dog genome for ATR1 and ATR2 and that are expressed in mouse brain.

Figure S1. Probabilities of changes in the allele frequencies occurring in a colony of 16 pairs of heterogeneous stocks.

Figure S2. Contributions of one-strain-specific SNPs in the WHS and the results of selection mapping for the control groups.

Figure S3. The haplotype derived from the MSM strain was not selected ingroup C1 at generation G_{12}.

Figure S4. Genome-wide heterozygosity in C1 (a) and S2 (b) groups.

Movie S1. Supplementary movie showing a typical example of a mouse in selected group S2.

Movie S2. Supplementary movie showing a typical example of a mouse in control group C1.

Table S1. Origins of the WHS founder strains and the 54 strains used in the phylogenic analysis

Strain	Abbr.	Subspecies group	Place of collection	Year of capture
BFM/2Ms	BFM/2	*M.m.domesticus*	Montpellier, France	1976
PGN2/Ms	PGN2	*M.m.domesticus*	Ontario, Canada	1979
HMI/Ms	HMI	*M.m.castaneus*	Heimei, Taiwan	1986
BLG2/Ms	BLG2	*M.m.musculus*	General Toshevo, Bulgaria	1980
CHD/Ms	CHD	*M.m.musculus*	Chendu, China	1981
KJR/Ms	KJR	*M.m.musculus*	Kojuri, Korea	1984
MSM/Ms	MSM	*M.m.musculus*	Mishima, Japan	1978
NJL/Ms	NJL	*M.m.musculus*	Northern Jutland, Denmark	1980

List of 54 strains used for NJ tree analysis

AEJ/GnLeJ, BFM2/Ms, A/J, AKR/J, BALB/cJ, C3H/HeJ, C57BL/6J, DBA/2J, I/LnJ, RIIIS/J, BLG2/Ms, 129S1/SvImJ, CAST/EiJ, NOD/ShiLtJ, NZO/HILtJ, PWK/PhJ, WSB/EiJ, CHD/Ms, CZECHI/EiJ, CZECHII/EiJ, DDY/Jcl, FVB/NJ, FVB, HMI, JE/LeJ, JF1/Ms, KJR/Ms, KK/HIJ, LEWES/EiJ, LG/J, LP/J, LT/SvEiJ, MOLF/EiJ, MRL/MpJ, MSM/Ms, CBA/CaJ, LP/J, NJL/Ms, NZW/LacJ, P/J, PERC/EiJ, PGN2/Ms, PWD, RBB/DnJ, RBF/DnJ, SF/CamEiJ, SH1/LeJ, SOD1/EiJ, ST/bJ, SWR/J, TIRANO/EiJ, WLA/Pas, WMP/Pas, ZALENDE/EiJ

Table S2. Threshold for each strain-specific SNP after Bonferroni correction and the observed maximum allele frequencies for the eight founder strains in the four WHS groups

Strain		PGN2	BFM/2	HMI	BLG2	CHD	NJL	MSM	KJR
# of SNPs		8,109	6,038	4,533	724	585	275	158	108
C1	Threshold	0.797	0.797	0.797	0.750	0.734	0.766	0.750	0.719
	Observed	0.625	0.500	0.625	0.500	0.406	0.406	0.422	0.422
C2	Threshold	0.813	0.797	0.797	0.781	0.750	0.781	0.750	0.734
	Observed	0.594	0.484	0.578	0.422	0.422	0.563	0.484	0.500
S1	Threshold	0.781	0.781	0.781	0.750	0.719	0.750	0.734	0.703
	Observed	0.578	0.531	0.563	0.484	0.516	0.516	0.375	0.391
S2	Threshold	0.781	0.797	0.781	0.766	0.750	0.766	0.734	0.734
	Observed	0.547	0.516	0.531	0.547	0.484	0.594	**0.750**	0.344

Table S3. Profiles of 23 mouse genes in the syntenic regions of the dog genome for ATR1 and ATR2 and which are expressed in mouse brain

Region	Gene	Start position (bp)	
		Mouse	Dog
Ⅰ	*Slc6a4*	76,812,105	47,549,263
Ⅱ	*Zfp207*	80,196,781	43,916,907
Ⅱ	*Psmd11*	80,242,117	43,829,963
Ⅱ	*Cdk5r1*	80,290,548	43,823,095
Ⅲ	*Dhx58*	100,556,018	24,104,700
Ⅲ	*Kat2a*	100,566,060	24,096,480
Ⅲ	*Rab5c*	100,576,323	24,089,152
Ⅲ	*Hcrt*	100,622,383	24,047,856
Ⅲ	*Stat5b*	100,642,045	24,008,792
Ⅲ	*Stat5a*	100,720,665	23,934,905
Ⅲ	*Stat3*	100,746,412	23,899,353
Ⅲ	*Ptrf*	100,818,047	23,828,660
Ⅲ	*Atp6v0a1*	100,870,766	23,740,530
Ⅲ	*Coasy*	100,943,879	23,709,182
Ⅲ	*Fam134c*	100,957,636	23,681,243
Ⅲ	*Ezh1*	101,052,429	23,589,579
Ⅲ	*Ramp2*	101,107,342	23,568,554
Ⅲ	*Becn1*	101,147,266	23,522,490
Ⅲ	*G6pc*	101,228,875	23,450,827
Ⅲ	*Rundc1*	101,286,399	23,390,295
Ⅲ	*Rnd2*	101,326,313	23,355,999
Ⅲ	*Brca1*	101,350,078	23,278,875
Ⅲ	*Nbr1*	101,413,463	23,246,150

a. Probabilities of loss of alleles under neutrality

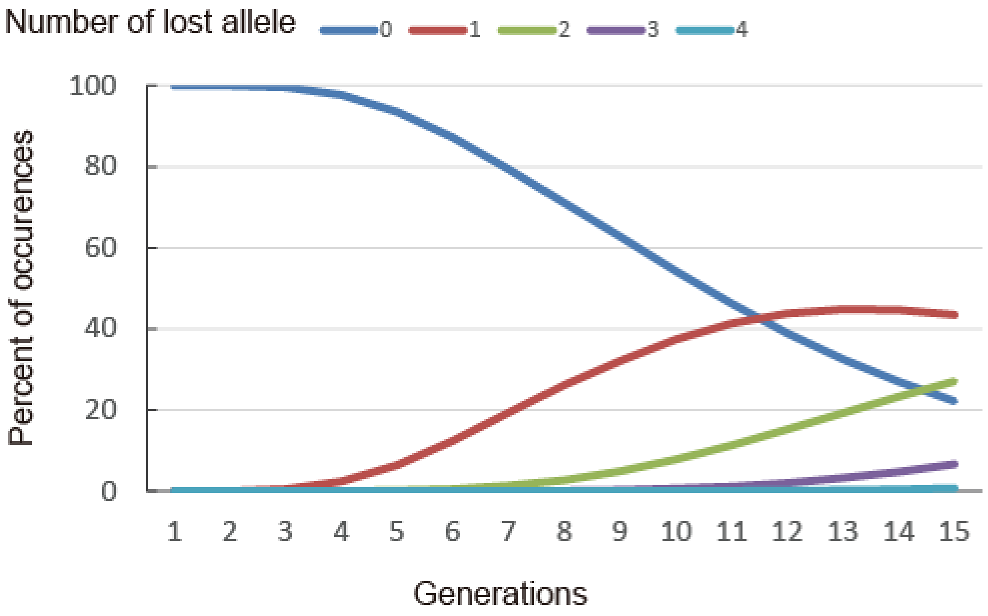

b. Expected distributions of the copy number of an allele under neutrality

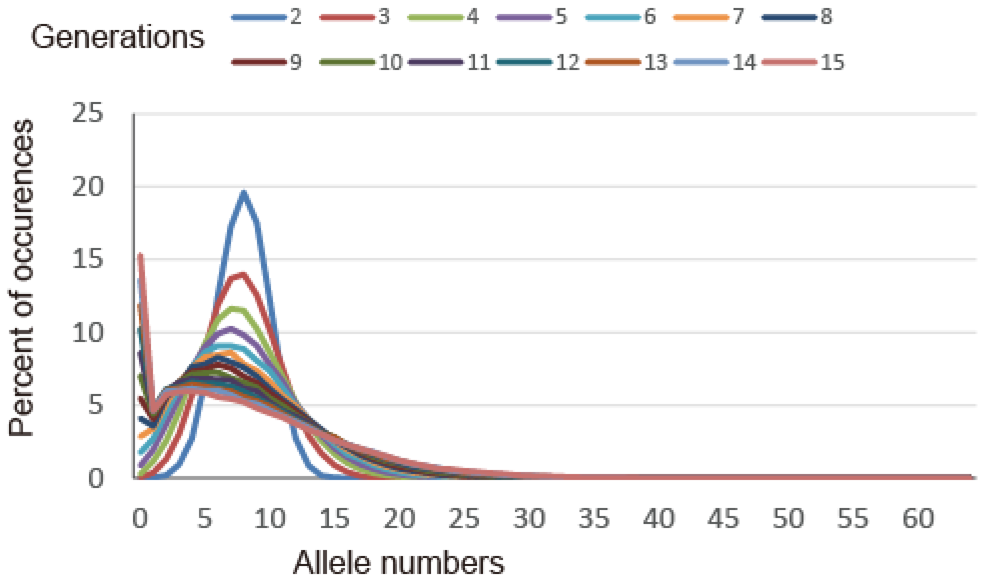

Figure S1. Probabilities of changes in the allele frequencies occurring in a colony of 16 pairs of heterogeneous stocks. The stocks were kept using a circle breeding design, and there were initially eight copies of each of the eight alleles at each locus. All data were obtained from 100,000 simulations. (a) Probabilities that alleles were lost under neutrality. (b) Expected distributions of the copy numbers of the 64 alleles (16 pairs of mice) under neutrality.

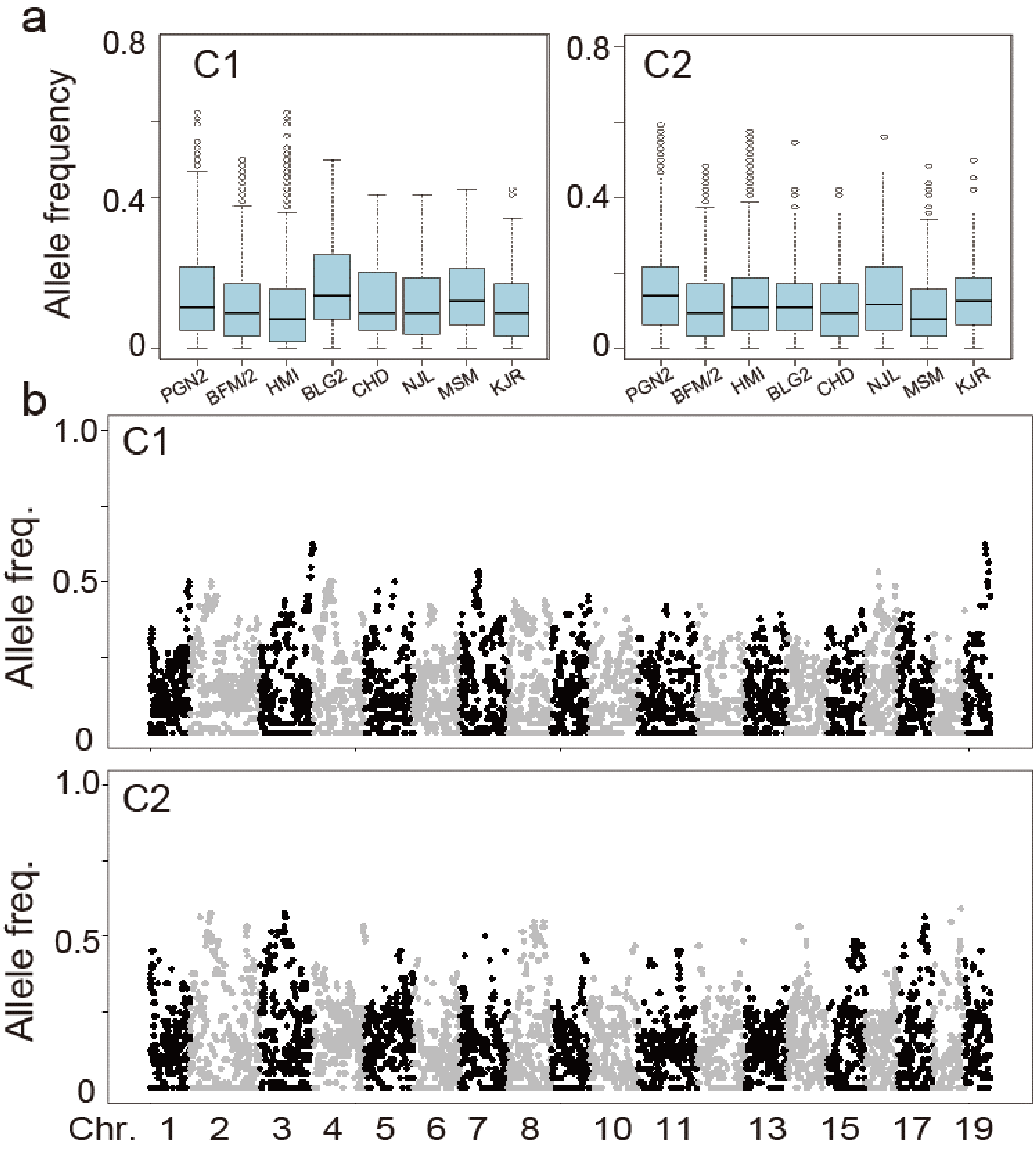

Figure S2. Contributions of one-strain-specific SNPs in the WHS and the results of selection mapping for the control groups. (a) Genome-wide average frequencies of 20,530 one-strain-specific SNPs in groups C1 and C2 at generation G_{12}. (b) Results of selection mapping in groups C1 and C2. No SNP reached the threshold determined using the computer simulation described in Table S2 in either group C1 or group C2.

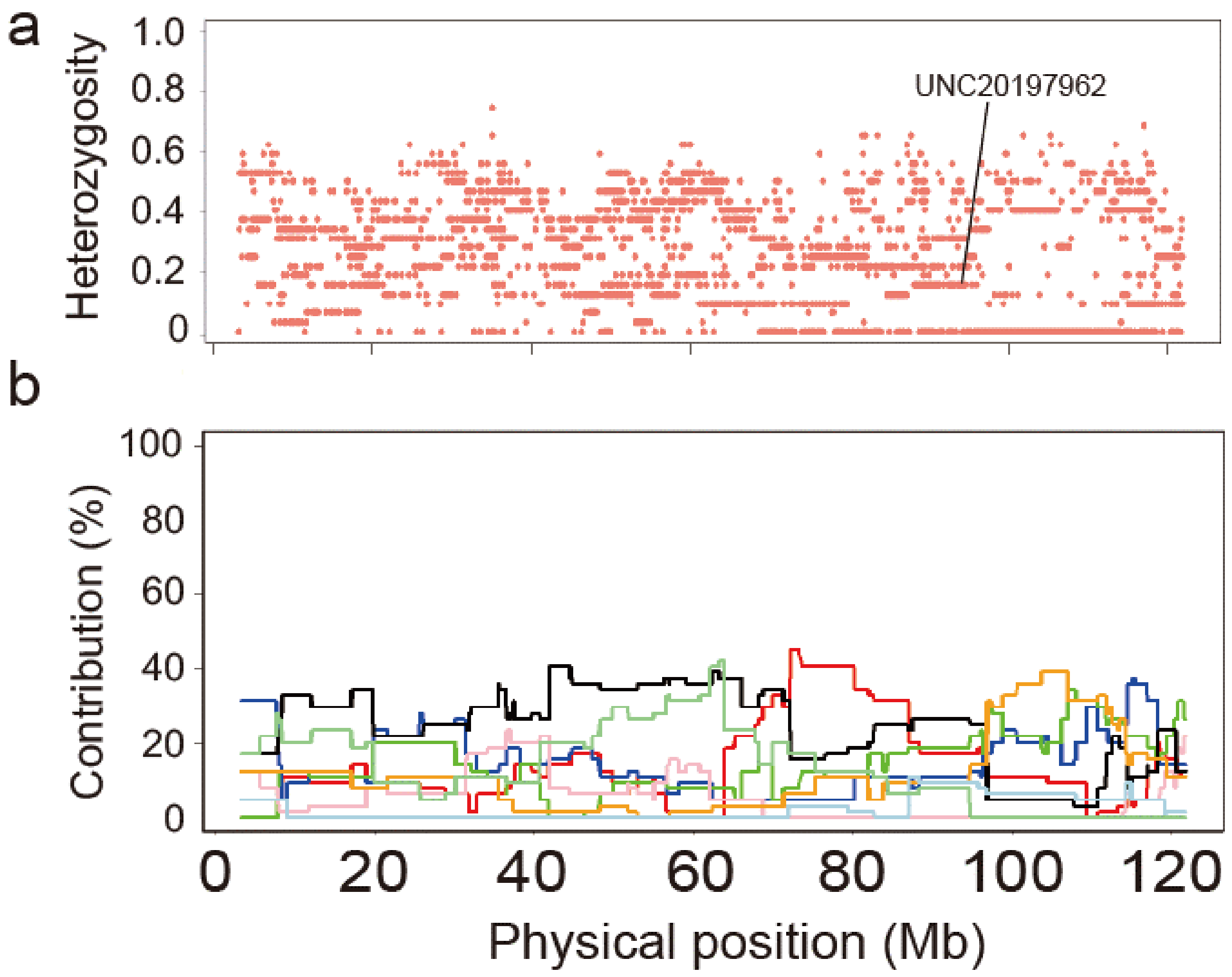

Figure S3. The haplotype derived from the MSM strain was not selected in group C1 at generation G_{12}. (a) Heterozygosity determined with 100 SNPs sliding windows with 10 SNPs gap. (b) Contributions of inferred haplotypes from the eight founder strains.

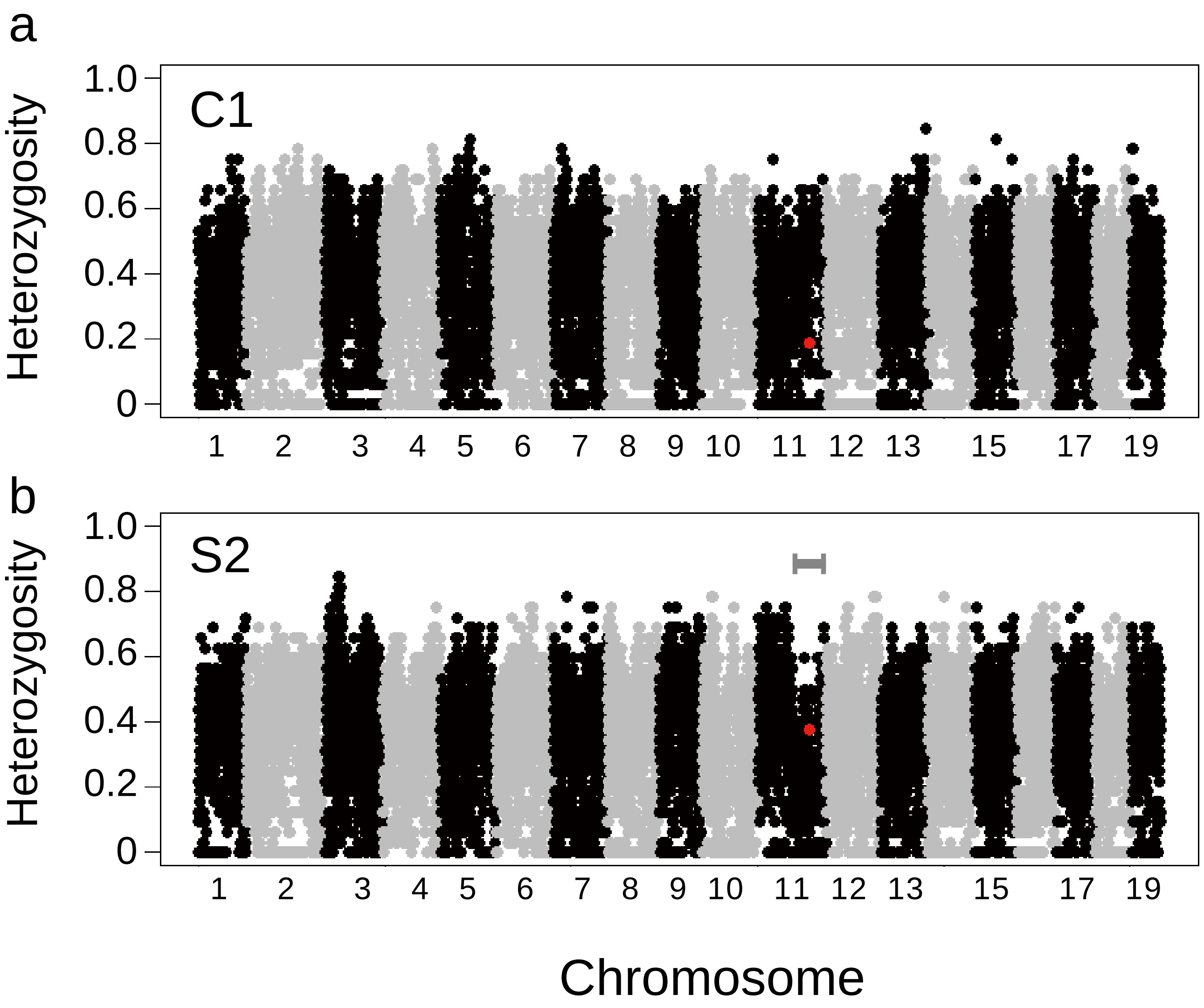

Figure S4. Genome-wide heterozygosity in C1 (a) and S2 (b) groups. The red dot on Chromosome 11 indicates detected SNP (UNC20197962) by using the simulation based analysis. The solid horizontal lines indicates the region showing decrease of the heterozygosity. The estimated heterozygosity of the SNP (UNC20197962) was greater in S2 group (H =0.375) than that of C1 group (H =0.1875). This result can be explained by the Hardy-Weinberg equation for considering the allele frequencies of SNP (UNC20197962) for S2 and C1 were 0.75 and 0.188, respectively. Based on the Hardy-Weinberg equation, heterozygosity increase as the allele frequency increase. The heterozygosity will reach the maximum value at the point when allele frequency is 0.5. However, the heterozygosity will decrease when the allele frequency exceeds the 0.5. According to the theory, the allele frequency of UNC20197962 in S2 is 0.75, then the theoretical heterozygosity is 0.375 (and actual heterozygosity is 0.375 in our data). By contrast, the allele frequency in C1 is 0.188, then the theoretical heterozygosity is 0.305 (but actual heterozygosity is 0.188 in our data).
